# Glargine and degludec: Solution behaviour of higher dose synthetic insulins

**DOI:** 10.1038/s41598-017-06642-w

**Published:** 2017-08-04

**Authors:** Gary G. Adams, Qushmua Alzahrani, Shahwar I. Jiwani, Andrew Meal, Paul S. Morgan, Frank Coffey, Samil Kok, Arthur J. Rowe, Stephen E. Harding, Naomi Chayen, Richard B. Gillis

**Affiliations:** 1 The University of Nottingham, Faculty of Medicine and Health Sciences, Queen’s Medical Centre, Nottingham, NG7 2UH UK; 2 The University of Nottingham, School of Biosciences, National Centre for Macromolecular Hydrodynamics (NCMH), Sutton Bonington Campus, Sutton Bonington, Leicestershire LE12 5RD UK; 3 Abant Izzet Baysal University, Faculty of Engineering & Architecture, Department of Food Engineering, Gölköy Bolu, Turkey; 4 Imperial College London, Faculty of Medicine, Department of Surgery & Cancer, Sir Alexander Fleming Building, South Kensington Campus, London, UK

## Abstract

Single, double and triple doses of the synthetic insulins *glargine* and *degludec* currently used in patient therapy are characterised using macromolecular hydrodynamic techniques (dynamic light scattering and analytical ultracentrifugation) in an attempt to provide the basis for improved personalised insulin profiling in patients with diabetes. Using dynamic light scattering and sedimentation velocity in the analytical ultracentrifuge glargine was shown to be primarily dimeric under solvent conditions used in current formulations whereas degludec behaved as a dihexamer with evidence of further association of the hexamers (“multi-hexamerisation”). Further analysis by sedimentation equilibrium showed that degludec exhibited reversible interaction between mono- and-di-hexamer forms. Unlike glargine, degludec showed strong thermodynamic non-ideality, but this was suppressed by the addition of salt. With such large injectable doses of synthetic insulins remaining in the physiological system for extended periods of time, in some case 24–40 hours, double and triple dose insulins may impact adversely on personalised insulin profiling in patients with diabetes.

## Introduction

The ideal insulin profile for any individual with diabetes will fluctuate due to lifestyle variations and metabolic influences – for example hypoglycaemia - so the physiological importance of insulin is vital for glycaemic homeostasis^1^. The biologically active, circulating form of insulin is monomeric in structure and consists of two chains, an A chain of 21 amino acids and a B chain of 30 amino acids (human), linked by two disulfide bridges, A7–B7 and A20–B19. The A chain contains an intra-chain disulfide bridge between A7 and A11. In the presence of zinc ions and at micromolar concentrations, insulin has been shown to dimerise and further associate into hexamers. Hodgkin and colleagues’ pioneering work of the’ 2-Zn hexamer^2^ showed the A chain has an N-terminal helix (A1–A8) linked to an anti-parallel C-terminal helix (A12–A20)^3^. The B chain has a central helix (B8–B19), which is extended by N- and C terminal strands. This is referred to as the crystallographic T conformation, where all six monomers are in the T (T6) conformation. An alternative conformation where the B-chain helix extends all the way to the N-terminal (B1–B19) is referred to as the R conformation.

The 4-Zn hexamer discovered by Schlichtkrull^4^ and generated by high chloride concentrations showed three of the monomers were in the R form and three in the T form (R3T3), a structure eventually solved by Hodgkin’s group. All six monomers are in the R form (R6) in phenol-containing crystals^5^. The R6 hexamer (solution structure) was resolved by NMR and the allosteric equilibrium structure (T–R transition) was also resolved, where it plays an important role in the pharmaceutical formulations of insulin, whereby phenol and chloride are used as antimicrobial and isotonic agents, respectively.

It has now been established that the basic insulin structure of three helices with three conserved disulfide bridges is present in all members of the insulin peptide family. Around its hydrophobic core, the insulin monomer has two extensive non-polar surfaces, one of which is flat and mainly aromatic, and concealed on dimer formation in an anti-parallel beta sheet and the other is more extensive and is hidden when the dimer to hexamer transition occurs^6^.

With this in mind, physiological *normoglycaemia* is modulated by the islet *β*-cell response to lower or higher plasma glucose concentrations.

In Diabetes Mellitus (DM), normal physiological processes do not occur and chronic hyperglycaemia is apparent, where glycaemic control is severely impaired as a result of a deficiency in insulin or its action. This deficit induces metabolic and degenerative microvascular and macrovascular complications in multiple organs including the heart, nerves, eyes and kidneys. Historically, bovine and porcine insulins have been used to treat diabetes mellitus.

In the 1930s and 1940s, Neutral Protamine Hagedorn (NPH insulin) and zinc-based products extended the action of unmodified insulin, but neither NPH nor the zinc (Lente) series attained the requisite flat insulin delivery profile, representative of peak plasma insulin concentrations approximately 5 h post-administration with diminishing insignificant levels by 10–12 h. Such physiological profiles are incongruous with high insulin sensitivity at night, or rising prerequisites at dawn with resulting consequences of nocturnal hypoglycaemia and pre-breakfast hyperglycaemia. The only other treatments available for combatting ineffective endogenous insulin production are the synthetic insulins and analogous structures. New basal insulins with longer and flatter profiles were designed from 1970 to 2000 such as highly purified bovine *ultralente* insulin. Unfortunately, due to erratic absorption and poor bioavailability these insulins were unsuccessful. In 1995, some success was gained with soluble insulins, *in vitro*, at acidic pH with microprecipitation at neutral pH in tissues (insulin *glargine*) and those derivatised with a fatty acid moiety to promote albumin binding, consequently delaying absorption (insulin *detemir*). Although, both provide night-time coverage, and insulin detemir’s shorter absorption profile may be compensated for by the intravascular albumin binding buffer erratic changes in insulin absorption, neither is a true 24-h insulin in people with type 1 diabetes. Insulin *degludec*, an acylated analogue of human insulin has an absorption profile which is longer than 24 h, with a half-life of ∼25 h^7, 8^.

The pharmaceutical companies who manufacture these chemical formulations are constantly altering the structural integrity of these synthetic insulins in an attempt to achieve more physiological replacement therapies, with consequentially improved glycaemic control. These synthetic insulins, however, possess structural modifications that may impact adversely on patients.

Despite some improvements in blood glucose control, factors such as user convenience, side effects, in addition to mean glucose control remain poor in clinical practice, and the challenge of hypoglycaemia continues to be problematic.

Many studies have examined different synthetic insulins using a range of different techniques^9–13^. However, in an attempt to understand further the structural characteristics of each synthetic insulin in relation to their relative performance, we have characterised for the first time, single, double and triple doses of synthetic insulins using macromolecular hydrodynamic techniques.

## Results

### Dynamic Light Scattering

**Figure 1:**
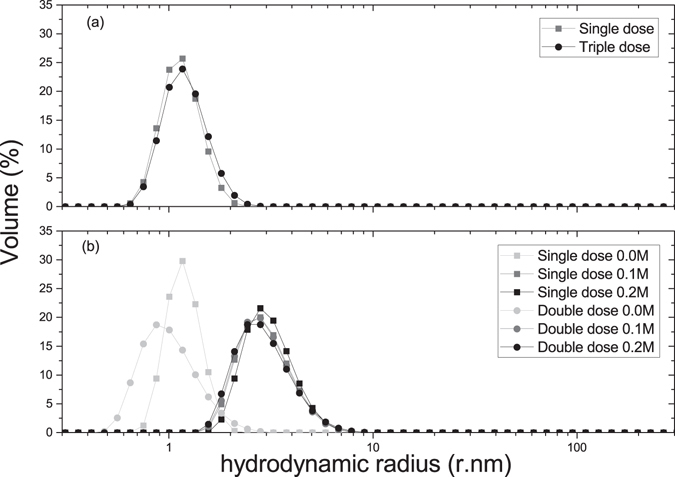
Volume (%) against hydrodynamic radius of insulin obtained from dynamic light scattering of (**a**) Insulin glargine (single and triple dose) and (**b**) Insulin degludec (single and double dose at 0.0, 0.1 and 0.2 M ionic strength).

Figure 1 shows a comparison of distributions of hydrodynamic radii estimated by dynamic light scattering^14^ as a proportion of the volume in solution. Insulin glargine single dose (IGS) and IGT (triple dose) show matching distributions which equate to hydrodynamic radii of (1.3 ± 0.1) and (1.4 ± 0.1) nm, respectively.

Insulin degludec single dose (IDS) and IDD (double dose), at unmodified ionic strengths to those of the commercial product, show statistically significantly different distributions (Z test, p ≪ 0.05). The peaks (Fig. 1b) both show hydrodynamic radii of (1.2 ± 0.1) nm. Upon modification with ionic strength, all four samples (single/double dose, 0.1/0.2 M) fall upon the same distribution. IDS 0.1 M showed (3.1 ± 0.3) nm, IDS 0.2 M showed (3.2 ± 0.3) nm, IDD 0.1 M showed (3.0 ± 0.3) nm and IDD 0.2 M also showed (3.0 ± 0.3) nm. Table 1 compares the values and the corresponding translational diffusion coefficients.

**Table 1:** Hydrodynamic radii and corresponding diffusion coefficients of insulins glargine and degludec, measured using dynamic light scattering.

		Hydrodynamic radius (r.nm)	Diffusion coefficient (x10^7^ cm^2^/s)
Glargine	Single dose 0.0 M	1.3 ± 0.1	14 ± 1
	Triple dose 0.0 M	1.4 ± 0.1	14 ± 1
Degludec	Single dose 0.0 M	1.2 ± 0.1	15 ± 2
	Single dose 0.1 M	3.1 ± 0.3	6.0 ± 0.6
	Single dose 0.2 M	3.2 ± 0.3	5.8 ± 0.6
	Double dose 0.0 M	1.2 ± 0.1	16 ± 2
	Double dose 0.1 M	3.0 ± 0.3	6.3 ± 0.6
	Double dose 0.2 M	3.0 ± 0.3	6.3 ± 0.6

### AUC-SV

**Figure 2:**
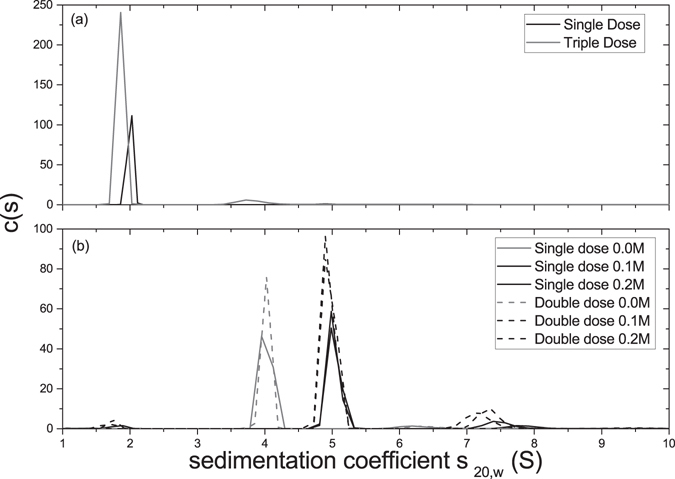
The sedimentation coefficient distribution c(*s*) plotted against sedimentation coefficient *s* (corrected for solvent conditions) for insulin samples obtained through AUC-SV. (**a**) Insulin glargine; (**b**) Insulin degludec at 0.0, 0.1 and 0.2 M ionic strength.

Figure 2 represents the sedimentation patterns (sedimentation coefficient distributions, c(s) vs s – see Dam and Schuck^15^) for the insulin preparations.

IGS and IGT both presented major peaks at approximately 2 S. Specifically, IGS had a sedimentation coefficient of (2.0 ± 0.1)S (96% of macromolecular material, which is considered of high purity) and IGT with two peaks at (1.9 ± 0.1)S (93%) and (3.7 ± 0.2)S (7%).

IDS and IDD show generally larger sedimentation coefficients than IGS and IGT. At 0.0 M (unmodified) ionic strength – i.e. no added salt - the major peaks appear at (4.2 ± 0.4)S (89%) and (4.0 ± 0.4)S (93%) for IDS and IDD, respectively. Faster-sedimenting peaks were also present at ~7 S (IDS 5.5%, IDD 3.7%) and ~11 S (IDS 4.2%, IDD 3.2%, not shown). At higher ionic strength, the major peak shifted from 4 S to 5 S for both concentrations and both ionic strengths. IDS 0.1/0.2 M yielded sedimentation coefficients of 5.3 S (86%/94%) and IDD 0.1/0.2 M yielded (5.0 ± 0.5)S (82%/83%).

### AUC-SE

Sedimentation equilibrium profiles were analysed according to two relevant analysis routines: INVEQ^16^ and MULTISIG^17^. INVEQ is able to provide estimates of the second virial coefficient; and MULTISIG provides low-resolution distributions of molar masses.

**Figure 3:**
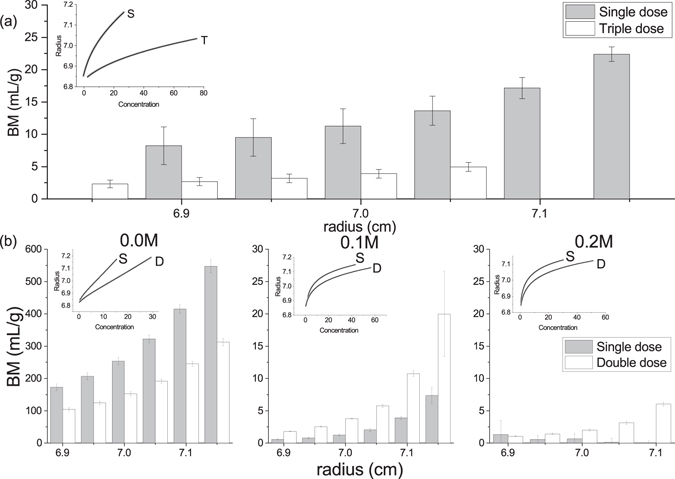
*BM* estimated by the INVEQ algorithm of insulin preparations. (**a**) glargine AUC-SE profile measured at 25 000 rpm (inset, S = single dose, T = triple dose), with the second virial coefficient factor *BM* fitted at different radial positions for single (grey) and triple (white) dose. (**b**–**d**) Degludec measured at 15 000 rpm (insets, S = single dose, D = double dose), with (**b**) unmodified ‘0.0 M’ ionic strength, (**c**) 0.1 M ionic strength; and (**d**) 0.2 M ionic strength. *BM* was fitted at different radial positions for single (grey) and double (white) dose. Standard error of regression indicated with error bars which represents how closely the fit matches the raw data. Note the 30x difference between the *BM* scales of (**b**) and (**c**,**d**).

Figure 3 represents the output from the sedimentation equilibrium analysis routine INVEQ over different radial points in the AUC-SE dataset (see inset).

The error bars in Fig. 3 represent the standard error of regression. Consequently, IGS fitted *BM* values between 8 and 23 mL/g, whereas IGT fitted between 2 and 5 mL/g.

IDS and IDD (unmodified by ionic strength) show orders-of-magnitude greater BM values than glargine; between 180 and 550 mL/g for single dose and between 100 and 300 mL/g for double dose. The pattern of higher dose fitting lower *BM* values is the same between both glargine and degludec datasets. With the addition of ionic strength, *BM* values are reduced to 1–20 mL/g levels, similar to glargine. The inset SE traces are also visibly different to the unmodified degludec traces. In fact, IDS 0.2 M fitted close to 0 mL/g, although IDD 0.2 M gave more expected results.

**Figure 4:**
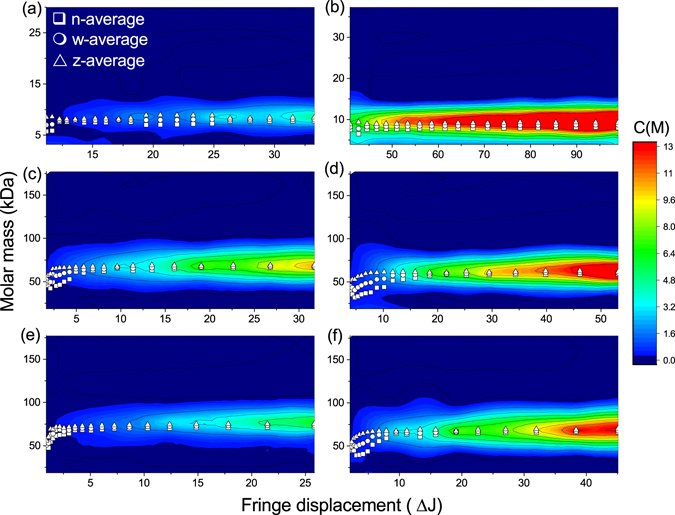
Molar mass as a function of concentration (expressed in fringe displacement units), with number (☐), weight (◯) and z (Δ) averages. Darker contours represent higher proportion of solute in solution. (**a**) IGS; (**b**) IGT; (**c**) IDS 0.1 M; (**d**) IDD 0.1 M; (**e**) IDS 0.2 M; (**f**) IDD 0.2 M.

Figure 4(a) and (b) represent glargine at single and triple dose, respectively. Degludec is represented from (c-f) where (c) and (d) are 0.1 M at single and double dose; and (e) and (f) are 0.2 M at single and double dose respectively.

The increase in dosage for (b) is reflected by the higher contours (concentration, C(*M*), where peak height is the concentration). Throughout the cell, the molar mass is represented at ~10 kDa. There is also only 1 peak present in both doses throughout the cells. Number average, weight average and z-average molar molar masses (*M*_n_, *M*_w_, *M*_z_ respectively) for IGS were 7.97 (±0.12%), 8.06 (±0.06%) and 8.15 (±0.01%) kDa (±standard error of mean). Both z/w and w/n polydispersity indices (PDI) were low at 1.01. IGT yielded similar averages: 8.08 (±0.17%), 8.67 (±0.09%) and 9.23 (±0.02%) kDa and z/w and w/n PDIs of 1.07.

Unmodified IDS and IDD could not be analysed using the MULTISIG/RADIUS method because of the very high thermodynamic non-ideality. With the presence of added salt, both dosages were analysable and are presented in 4 (c-f). As with glargine, the increase in dose (d, f) is indicated by a higher, darker contour on the plot. Number, weight and z-average molar masses were 65.5 (±0.42%), 67.1 (±0.17%) and 68.6 (±0.04%) kDa for IDS 0.1 M; 58.4 (±0.37%), 60.2 (±0.12%) and 61.8 (±0.08%) kDa for IDD 0.1 M; 71.0 (±0.15%), 73.2 (±0.04%) and 74.8 (±0.03%) kDa for IDS 0.2 M; 66.1 (±0.32%), 67.0 (±0.09%) and 67.8 (±0.12%) kDa for IDD 0.2 M. IDS0.1 (c) shows evidence of a sigmoidal curve originating at 50 kDa close to the column meniscus (2.5 J, ≡ 6.85 cm) and increasing to 65 kDa by the cell base. At a fringe increment of around 4 (6.91 cm) the polydispersity temporarily increases but reduces again by 6 (6.97 cm). This pattern is reflected in all four degludec analyses.

### SINGLEHYDFIT

**Figure 5:**
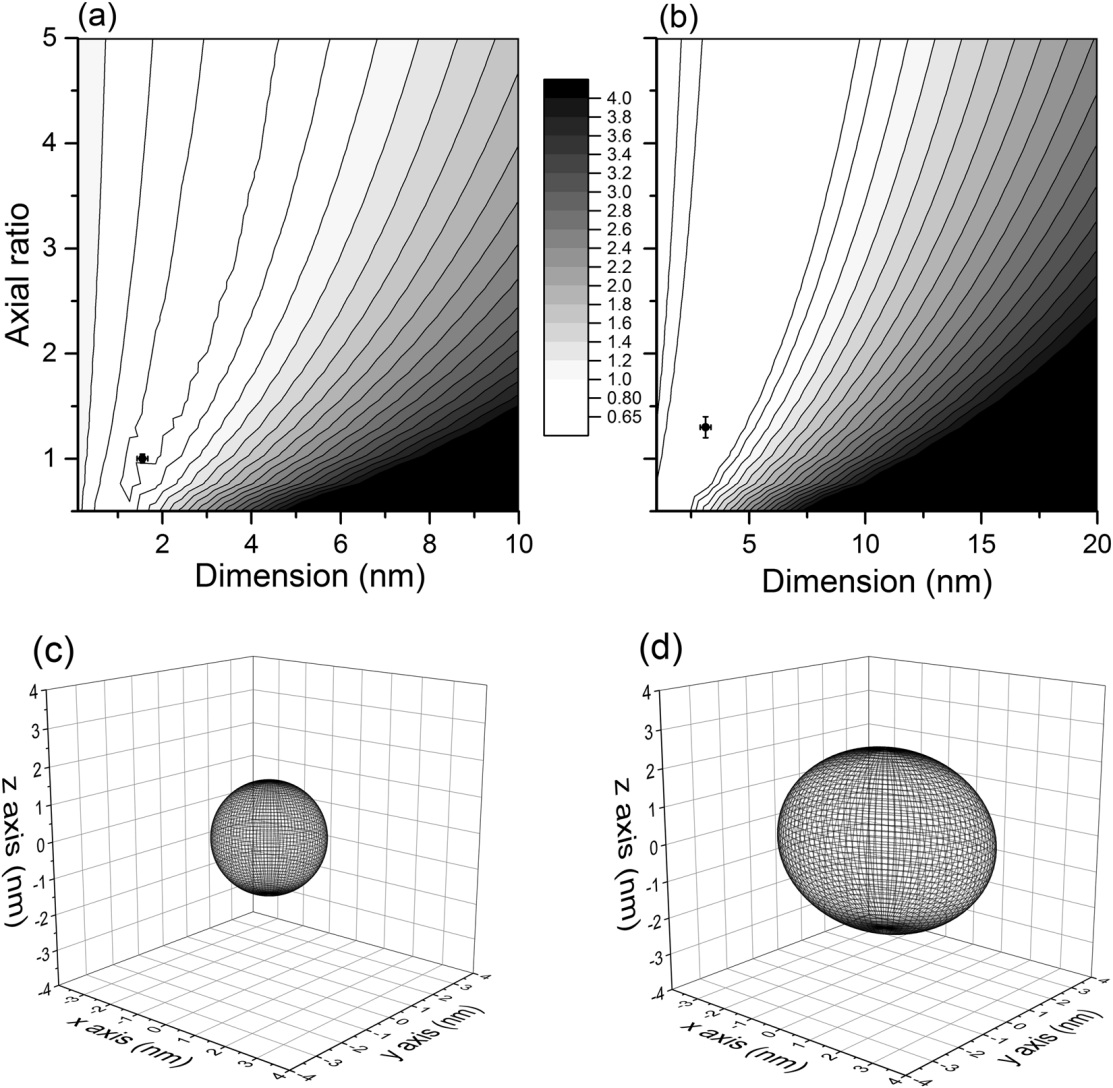
(**a**) and (**b**); contour probability distributions of glargine and degludec (respectively) from SINGLEHYDFIT where low Δ represents the lowest error of parameters. (**c**) and (**d**); to-scale ellipsoid representations of glargine and degludec (respectively) as fitted from SINGLEHYDFIT.

Data were combined, including diffusion coefficient, sedimentation coefficient, second virial coefficient, partial specific volume and molar mass to provide information in terms of size and axial ratio using the program SINGLEHYDFIT^18^. The time averaged hydration of the insulins was estimated at ~0.34 g/g (mass of water per mass of protein solute) from SEDNTERP^19^: this helped to refine the fitting search. Output of this analysis is shown in Fig. 5, the analysis for which will be presented in the discussion.

The combined information from DLS, AUC-SV and AUC-SE for glargine provided a best-fit local minimum at radius (1.6 ± 0.1) nm and an axial ratio of ~(1.0 ± 0.1), consistent with a dimer. In essence, this is close to a spherical conformation representing two monomers bound together.

Combined data for degludec suggested that this analogue forms predominantly a dodecamer, or di-hexamer. The fit provided local minima of (3.1 ± 0.3) nm and an axial ratio (prolate ellipsoid model) of (1.3 ± 0.1). The 3D model therefore represents two hexamers bound together, although this analysis cannot distinguish whether two hexamers sit on-top of each other (aligned along the z-axis), or side by side (aligned along the x-axis).

## Discussion

The long-acting recombinant basal synthetic insulins glargine and degludec were developed to overcome some of the disadvantages of early intermediate-acting basal insulin complexed with protamine (neutral protamine Hagedorn [NPH] insulin)^20, 21^.

Glargine and degludec (both available as U100 insulin or 100 U/mL) were modified to deliver more prolonged and stable pharmacokinetic and pharmacodynamic characteristics compared to NPH insulin: 1) a protracted duration of action, permitting once-daily dosing, 2) a reduction in clinically important rates of hypoglycaemia (including nocturnal and severe hypoglycaemia), and 3) lower within- and between-subject variability, leading to more consistent and predictable glycaemic control^10, 20, 21^.

In an attempt to understand further the structural characteristics of insulin in different dose forms and how these could potentially impact on physiological function, our research group characterised for the first time, single, double and triple doses of synthetic insulins, glargine and degludec.

### Stoichiometry

Stoichiometry refers to the quaternary structure of proteins in solution, i.e. their solution state as a monomer, dimer, trimer etc. Glargine showed consistent evidence of being predominantly in dimer conformation in both single and triple dose preparations. This is posited from the combination of hydrodynamic parameters from dynamic light scattering (DLS) and Analytical Ultracentrifugation (AUC). At the higher dose (triple concentration), however, sedimentation velocity (AUC-SV) showed evidence of higher n-mer stoichiometries such as a hexamer at 3.7 S, which is consistent with current knowledge of protein binding kinetics. An increase in total concentration will prompt a change in equilibrium to increase intermolecular binding^22^. With this in mind, it has been postulated that certain analogues, namely glargine, may display insulin-like growth factor (IGF1-like) activities^23–25^. The IGFs constitute a network of ligands, cell-surface receptors, and binding proteins with important roles in cell cycle progression^26^. It has also been postulated that these analogues, by acting as insulin-like growth factors, potentially initiate tumourigenesis^27^. Since one of the main reasons insulin analogues were designed was a protracted duration of action, permitting once-daily injection, and these analogues spend longer *in vivo*, it is reasonable to suggest that glargine may be having an even greater effect on cell cycle progression than previously thought.

In addition, our research has shown that increased concentration increases self-interaction affinity due to a change in equilibrium kinetics. It is also reasonable to suggest that increased concentration also initiates an increased affinity for binding to all IGFs. Although our findings were not replicated by the sedimentation equilibrium (AUC-SE) analysis above, possibly due to the low-resolution nature of AUC-SE compared to SV and the relatively low proportion of hexamer estimated from AUC-SV, the fact that glargine may potentiate tumourigenesis requires more extensive studies to be carried out.

For degludec, AUC-SE analysis also showed a reversible equilibrium between a hexamer (~37 kDa) and di-hexamer (~73 kDa), although the hexamer was not present in AUC-SV analysis. This multi-hexameric behaviour of degludec, an engineered acylated insulin which forms a soluble depot after subcutaneous injection, is consistent with previous studies^28^ but this is the first time that different doses of degludec have been shown to promote multi-hexamerisation. This acylated insulin is an engineered des(B30) human insulin modified with a hexadecanoic diacid via a γ-L-glutamyl linker to Lys B29, and developed with the intention of forming a large soluble zinc complex in the sub-cutaneous layer. The main concerns here, however, are: 1) Whether this soluble depot of single and double dose degludec are absorbed in a similar fashion to human insulin; 2) Whether single and double doses behave in a similar physiological manner to each other; and 3) If a change in concentration impacts current clinical problems, such as hypoglycaemic unawareness^29^.

### Ionic strength and non-ideality

As an injectable insulin preparation, degludec does not contain any appreciable salt concentration other than zinc ions necessary for the stapling of monomers/dimers into hexamers. However, when assessed using hydrodynamic techniques, the unmodified protein solution provided challenges to analysis. The anomalously high diffusion coefficient for degludec at low ionic strength, *I*, appears to be due to the effects of non-ideality (see Harding & Johnson^30^ & Scott *et al*.^31^) through unsuppressed charge repulsion effects. Upon the addition of salt and increase of *I* to approximate physiological conditions, these electrostatic interactions are suppressed, reducing the apparent diffusion coefficient.

Degludec also yielded atypical AUC-SE concentration curves which proved impossible to analyse accurately using certain algorithms. It was only with the attainment of a physiological ionic strength that more typical curves were obtained. The INVEQ algorithm fitted 0.0 M data showing second virial coefficients 20 times greater than for both 0.1 M and 0.2 M. Therefore, the majority of this non-ideal behaviour can be attributed to unsuppressed charges at low I^32^. It is clear therefore that the ionic strength is having a significant impact on the behaviour of degludec. This has implications on the injection of this insulin into the subcutaneous layer and its subsequent behaviour as it crosses into the bloodstream.

Glargine, on the other hand, did not show levels of non-ideality higher than would be expected for a typical globular protein^33^. This is surprising as neither single nor triple dose formulations have a significant ionic strength, beyond zinc ions and hydrochloric acid. With a pH4 and an isoelectric point close to neutral, glargine ‘drops out’ of solution and it will be interesting to establish what physiological effect this has *in vivo*. Further investigations in our laboratory will elucidate whether, if any, there is an effect of ionic strength on hydrodynamic analysis of glargine.

Physiological Na^+^ molarity is 0.14 M^34^, which lies in-between the assessed ionic strengths of 0.1 and 0.2 M tested for degludec, has a pH7 and an isoelectric point of 5.5. However, other cations are present in the dermal layer, such as K^+^, Ca^2+^ and Mg^2+^ and need to be considered. There are also anions such as, Cl^−^, CO_3_^2−^, PO_4_^2−^, as well as more complex anions such as acetate, citrate, TRIS or HEPES. Although the assessment of the interactions between insulins and these various salts was not within the scope of this study, these findings do show that the ionic strength is critical to the formulation of these preparations, and that degludec may undergo conformational changes, which have not previously been identified, when injected into the physiological environment.

Glargine showed no significant difference between DLS distributions, but the triple dose concentration had a lower apparent diffusion coefficient (higher apparent hydrodynamic radius). The triple dose also had a lower apparent sedimentation coefficient. Since molar mass would not have changed, the decrease in sedimentation coefficient observed by AUC-SV through non-ideality would be linked through the decrease in diffusion coefficient, directly observed by DLS. The reduction in diffusion coefficient will have an effect *in vivo* in terms of glargine diffusion away from the subcutaneous depot.

Degludec, with unmodified ionic strength (i.e., as the injectable drug) has a very high degree of non-ideality from DLS with a statistically significant effect, however this has the effect of increasing the diffusion coefficient as explained previously with respect to valence and electrostatic interactions. AUC-SV, on the other hand, showed typical concentration dependence behaviour of slower-sedimenting peaks^30^.

With the addition of buffering salts, raising the ionic strength, AUC-SE analysis showed that the sigmoidal pattern changed with increase in degludec dosage. The polydispersity at the sigmoidal midpoint is increased and pushed further away from the meniscus. This is explained by the reaction kinetics between mono-hexamer and di-hexamer being pushed towards larger species as mentioned previously. This affects the rate of diffusivity, with potential physiological effects on the patient.

## Conclusion

In patients presenting with Diabetes Mellitus (DM), chronic hyperglycaemia is apparent where glycaemic control is severely impaired as a result of a deficiency in insulin or its action. Synthetic, exogenous insulins are injected subcutaneously to promote normoglycaemia.

In an attempt to understand further how certain insulins function and what characteristics of each relate to its performance, this research characterises for the first time, single, double and triple doses of synthetic insulin analogues, currently used in patient therapy using macromolecular hydrodynamic techniques.

Results showed that, using dynamic light scattering, glargine yielded a dimer and degludec a dihexamer (with added salt) and the increase of ionic strength appeared to removed concentration dependence non-ideality from degludec. In addition, sedimentation velocity characterisation demonstrated that glargine is predominantly a dimer, with degludec demonstrating a dihexamer conformation with evidence of multi-hexamerisation. By using the INVEQ algorithm (second virial coefficient), a significant degree of non-ideality of macromolecules was established, where the addition of salt reduced non-ideality of degludec by a factor of ~20. MULTISIG-RADIUS analysis of degludec exhibited reversible interaction between mono-and-di-hexamer conformations with SINGLEHYDFIT demonstrating dimer and di-hexamer stoichiometries.

With such large concentrations of injectable synthetic insulins remaining for extended periods of time in the physiological system, in some cases 24–40 hours, these may impact adversely on diabetes patients^27, 35^. Examples of where these may negatively impact the patient are hypoglycaemic unawareness and potentially aberrant cell cycle progression which leads to increased tumourigenesis. The next stage of our work is to establish structural changes, *in vivo*, which if identified, could then be used as a breakthrough template in rapidly designing better new insulins.

## Materials and Methods

Five established tests were chosen to characterise the two insulin doses, to provide a full spectrum of relevant parameters that had not previously been used to investigate the structural properties of synthetic insulins. A powerful complementary approach is analytical ultracentrifugation (AUC). An analytical ultracentrifuge is distinct from a conventional ultracentrifuge in that it has a specialised optical system(s) enabling the concentration distribution under the influence of strong centrifugal fields (up to 200 000 g) to be registered - and how that changes with time, which is important for insulin. The optical system most relevant in modern instrumentation (e.g., the Beckman XL-I), is the refractometric or Rayleigh Interference system which records concentration distributions as a function of radial displacement.

There are two main types of experiment: sedimentation velocity and sedimentation equilibrium. Sedimentation velocity provides us with information on the physical homogeneity of a sample, conformation and flexibility information - and an estimate of the molar mass distribution. In addition, provides us with interaction information if, for example, we assay for what is called ‘co-sedimentation’ phenomena (i.e. species sedimenting at the same rate as another)^36, 37^. In sedimentation equilibrium experiments, the sedimentation force due to the centrifugal field and the backforce due to diffusion are comparable. After a considerable period of time (usually 24 h), the two forces come to equilibrium and the concentration distribution remains constant: a sedimentation equilibrium experiment can provide absolute molar mass - primarily weight (mass) and z-averages and molar mass distribution.

### Phosphate-buffered conditions

2 M PBS was prepared using 1 M NaCl, 0.5 M KH_2_PO_4_ and 0.5 M Na_2_HPO_4_ dissolved in deionised water.

### Insulins

Insulin glargine single dose (IGS) and triple dose (IGT) were supplied by Sanofi Aventis and by the International Diabetes Trust (IDDT). Insulin degludec single dose (IDS) and double dose (IDD) were supplied by Novo Nordisk and by IDDT. Vials were kept refrigerated (4–8 °C) from arrival until use.

Sequences were obtained from patient information leaflets and, in combination with SEDNTERP^19^, used to yield monomer molar mass and partial specific volume. Glargine has a monomer mass of 6063 Da and partial specific volume of 0.728 mL/g. Degludec monomer is 6101 Da and partial specific volume 0.736 mL/g.

### Ionic strength adjustment

The ionic strength of degludec preparations was raised by gently stirring 3 mL of a preparation with a magnetic bead and adding 158 μL 2 M PBS (slowly, to prevent localised salting out) to raise the ionic strength to 0.1 M. A 1 mL aliquot was stored and refrigerated ( + 4–8 °C), and another 114 μL 2 M PBS was added to raise the total ionic strength to 0.2 M. The remaining solution was stored and refrigerated. This was performed for both single dose (IDS0.1/IDS0.2) and double dose (IDD0.1/IDD0.2) degludec. Aliquots were allowed to refrigerate overnight before experimentation.

### Density measurement

Samples were injected into an Anton Paar (Graz, Austria) DMA5000 oscillating capillary density meter. Injected insulin preparations were measured at 20.00 °C for their solution density. Deionised water was measured beforehand to confirm the calibration of the apparatus.

### Viscometry

Dynamic viscosity was measured using an automated micro-viscometer Anton Paar AMVn rolling ball viscometer equipped with a 1.6 d.mm silanised capillary and 1.5 d.mm stainless steel ball. Kinematic viscosity was measured as a function of the time taken for the ball to roll through the solution at 70° (n = 4), 60° (n = 4) and 50° (n = 6) angles, to account for non-Newtonian flow, at 20.00 °C and converted to the dynamic viscosity using the previously measured solution density. Calibration was checked prior to use using deionised water.

### Dynamic Light Scattering

DLS measurements were used to find the translational diffusion coefficient and hydrodynamic radius. Sterile samples were loaded into sterile, dust-free, acrylic plastic cuvettes and sealed to prevent dust contamination. Cuvettes were then loaded into a Malvern Zetasizer NanoZS (Malvern Instruments, Malvern, UK) and light scattering data obtained using Zetasizer software v6.2 or later. Measurements were performed at 20.00 °C at a scattering angle of 173° as the insulin samples were assumed to be close to spherical with no appreciable scattering angle-dependence, rendering extrapolation to zero angle unnecessary^14^. Solution viscosities, measured previously, were used to convert translational diffusion coefficients into hydrodynamic radii using the Stokes-Einstein equation:

1 $${r}_{H}=\frac{{k}_{B}T}{6\pi \eta {D}_{T}}$$

where *D*_*T*_ is the particle’s translational diffusion coefficient, while *T* is absolute temperature, *η* is the solution viscosity and *k*_*B*_ is the Boltzmann constant. Values are apparent values (i.e. not corrected for non-ideality^30^).

### Sedimentation Velocity

AUC-SV was performed on all samples at 45 000 rpm (~150 000 g) and (20.0 ± 0.1) °C in centrifuge cells constructed with 12mm path length, aluminium-filled epoxy centrepieces, sapphire windows and aluminium cell housing. Cells, loaded with 400 μL insulin preparation, were placed into a Beckman (Brea, CA, USA) Optima XL-I Analytical Ultracentrifuge. Rayleigh Interference fringe patterns were captured using ProteomeLab software v6 and converted into ASCII files of data consisting of radial distance from the centre of rotation (*r*) and fringe increment (*j*) at time point (*t*) for later analysis by SEDFIT v14 c(*s*) analysis^38^. Distributions of sedimentation coefficients (*s*_*T,b*_) were corrected to standard solvent conditions, namely the density and viscosity of water at 20.00 °C, to give (*s*_*20,w*_).

Sedimentation coefficients, *s* (unit Svedberg, S = 10^−13^ sec) are a function of the molar mass (*M*) and friction coefficient (*f*), as given by the Svedberg equation^39^:

2 $$s=\frac{M(1-\bar{v}\rho )}{{N}_{A}f}$$

where *N*_*A*_ is Avogadro’s constant (6.022 × 10^23^ mol^−1^), $\bar{v}$ is the partial specific volume (mL/g) and *ρ* is the solution density (g/mL). Values are apparent values (i.e. not corrected for non-ideality^30^).

### Sedimentation Equilibrium

AUC-SE was performed on the same instrument, software, cells and temperature as per AUC-SV. The rotor speeds for IGS/IGT were 30 000 rpm (~70 000 *g*) and for IDS/IDD were 15 000 rpm (~17 500 g) and samples were centrifuged until equilibrium was achieved (i.e. no net movement of solute) at these speeds. Cells were filled with a lower volume (120 μL) of insulin preparations (and reference solvent) to decrease the time to reach equilibrium.

Data were analysed using two algorithms. The MULTISIG-RADIUS algorithm^17^ was used to fit distributions of molar mass over the radius of the cell using the fitting software ProFit (QuantumSoft, Switzerland). An adaptation of the INVEQ algorithm^16^ was used to yield the second virial coefficient, a measure of non-ideality of a macromolecular solution, fitted using OriginLab (Northampton, MA, USA).

For INVEQ, the standard equation for non-ideal sedimentation equilibrium, which represents concentration as a function of radial displacement from the centre of rotation is inverted to give the form shown below, which represents radial displacement as a function of concentration:

3 $$r={((\mathrm{ln}(\frac{{c}_{r}}{{c}_{ref}})+0.5(\frac{\sigma }{1+2BM{c}_{r}}){r}_{ref}^{2})/(0.5(\frac{\sigma }{1+2BM{c}_{r}})))}^{0.5}$$

where *r* is the radial value at which the observed concentration *c*_*r*_ (after correction for baseline offset) is located; *c*_*ref*_ is the observed concentration at a defined radial reference position *r*_*ref*_ and *M* is the molar mass. *σ* is the weight-average molar mass reduced for flotation $(1-\bar{v}\rho )$, rotor speed (*ω*) and temperature (*T*):

4 $$\sigma =M(1-\bar{v}\rho ){\omega }^{2}/RT$$

In Equations 5 and 6, *B*, the second virial coefficient, indicates the concentration dependence of the apparent molar mass *M*_*w,app*_. *B* contains contributions from excluded volume (_*ex*_) and charge (Z). The exclusion volume contribution to B is defined by the excluded volume (*u*, mL), molar mass (*M*, Da) and Avogadro’s number (*N*_*A*_). *κ* and *r*_*s*_ are inverse screening length and solvated radius, as defined by Harding *et al*.^32^. Charge effects (*B*_*Z*_) can be reduced to insignificant levels through an excess of ionic strength (*I*).

5 $$\frac{1}{{M}_{w,app}}=\frac{1}{{M}_{w}}(1+2BMc+\ldots )$$

6 $$B={B}_{ex}+{B}_{Z}\,|\,{B}_{ex}=\frac{u{N}_{A}}{2{M}^{2}}\,|\,{B}_{Z}=\frac{{Z}^{2}}{4{M}^{2}I}(\frac{1+2\kappa {r}_{s}}{{(1+\kappa {r}_{s})}^{2}}+\ldots )$$

Analysis was also carried out using the MULTISIG algorithm^17^:

7 $${j}_{r}=\sum _{i=1}^{i=17}{c}_{i}{j}_{ref}{e}^{(0.5(0.5{\sigma }_{i}{1.15}^{(i-1)})({r}^{2}-{r}_{ref}^{2}))}+E$$

Where *j* is the fringe increment (the concentration as measured using Rayleigh Interference optics from AUC), uncorrected for baseline *E*, at radial position *r* or reference radial position *r*_ref_. *c*_*i*_ and *σ*_*i*_ represent relative concentration and sigma for iteration _i_. MULTISIG-RADIUS is where equation 7 is performed at various *r*_*ref*_ values.

### Statistics

Integration and peak analysis was performed using native algorithms in SEDFIT or using OriginLab. A Z-test for statistical significance between distributions was performed using Excel.
